# Children's activity and diet behaviours in the summer holidays versus school year

**DOI:** 10.1111/ijpo.13029

**Published:** 2023-03-21

**Authors:** Amanda Watson, Carol Maher, Rebecca Golley, Dot Dumuid, Alexandra Manson, Grant Tomkinson, Francois Fraysse, Tim Olds

**Affiliations:** ^1^ Alliance for Research in Exercise, Nutrition and Activity (ARENA), Allied Health and Human Performance, University of South Australia Adelaide South Australia Australia; ^2^ College of Nursing and Health Sciences, Flinders University Adelaide South Australia Australia

**Keywords:** child health, physical activity, sedentary behaviour, summer effects on weight gain

## Abstract

**Background:**

Evidence shows children gain more weight during the summer holidays versus the school year.

**Objectives:**

To examine within‐child differences in activity and diet behaviours during the summer holidays versus the school year.

**Methods:**

Children (mean age 9.4 years; 37% male) wore accelerometers (GENEActiv; *n* = 133), reported activities (Multimedia Activity Recall for Children and Adolescents; *n* = 133) and parents reported child diet (*n* = 133) at five timepoints over 2 years capturing school and summer holiday values. Mixed‐effects models were used to compare school and summer holiday behaviours.

**Results:**

Children spent less time in moderate‐ to vigorous‐physical activity (−12 min/day; *p* = 0.001) and sleep (−12 min/day; *p* < 0.001) and more time sedentary (+27 min/day; *p* < 0.001) during summer holidays versus the school year. Screentime (+70 min/day; *p* < 0.001), domestic/social activities (+43 min/day; *p* = <0.001), self‐care (+24 min/day; *p* < 0.001), passive transport (+22 min/day; *p* = 0.001) and quiet time (+16 min/day; *p* = 0.012) were higher during the summer holidays, compensating for less time in school‐related activities (−164 min/day; *p* < 0.001). Diet quality was lower (−4 points; *p* < 0.001) and children consumed fewer serves of fruit (−0.4 serves; *p* < 0.001) during the summer holidays versus the school year.

**Conclusions:**

Children are displaying poorer activity and diet behaviours during the summer holidays, which may contribute to accelerated weight gain over the holiday period.

## 1 BACKGROUND

A growing body of evidence indicates that more weight is gained during the summer holidays, compared with the school year.^1^, ^2^, ^3^, ^4^ This weight gain may be due to engagement in relatively more obesogenic behaviours (e.g., low levels of physical activity, high levels of screen time and poorer diet) during the summer holidays. A recent meta‐analysis of 296 studies of obesogenic behaviours on school days versus non‐school days (i.e., weekends of summer holidays) found that adolescents (12–19 years) engaged in lower levels of physical activity (SMD = −0.25) and more screen time (SMD = −0.48, reverse‐scored) on less structured days. Sleep timing (SMD = −1.05) and diet quality (SMD = −0.29) were also less healthy on less structured days.^5^ Most included studies^5^ examined differences in obesogenic behaviours on weekend days versus weekdays (*k* = 287), with just a few (*k* = 9)^6^, ^7^, ^8^, ^9^, ^10^, ^11^, ^12^, ^13^ comparing obesogenic behaviours during school holidays and school days, and only one specifically compared obesogenic behaviours in the summer holidays with school days.^14^ Using data from the National Health and Nutrition Examination Survey (NHANES) 2003–2008, Wang et al.^14^ found that US children (grades 1–12) surveyed during the summer holidays (*n* = 1339) were more active, watched more television, and consumed fewer vegetables and more added sugar than those surveyed during the school year (*n* = 5114). A key limitation of that study was the between‐subjects design, where children surveyed during the summer holidays were compared with a different group of children surveyed during the school year.^14^

Recent studies not included in the Zosel et al. meta‐analysis^5^ have used within‐subject study design to compare children's obesogenic behaviours in the summer holidays versus the school year. Volmut et al.^15^ reported overall physical activity decreased by 18%, moderate‐to‐vigorous physical activity (MVPA) decreased by 45%, while sedentary behaviour levels increased by 5.5% during summer holidays compared with before summer holidays among 93 Slovenian children aged 6–9 years. Similarly, Hunt et al.^16^ reported larger unhealthy changes in 267 Kindergarten to grade 4 US children's sedentary time, physical activity levels, sleep and screen time in the summer holidays versus the school year. While those studies provide insight into children's activity behaviours during summer versus school, neither assessed all obesogenic behaviours.

To our knowledge, only two studies have examined all obesogenic behaviours (activity behaviours and diet) in a single study using a within‐subject design.^17^, ^18^ Both found US children (predominantly low income, minority) spent more time sedentary (10–55 min/day) and slept longer (15 min/day in both studies) each day during the summer holidays compared with the school year. Results for physical activity and diet were mixed. Brazendale et al.^17^ reported children consumed more sugar‐based foods (6 days vs. 3.5 days/week) but MVPA did not differ during summer holidays compared with the school year. In contrast, Weaver et al.^18^ reported children engaged in less light‐intensity physical activity (LPA) (−42 min/day) and MVPA (−11 min/day) during summer holidays versus school but found no dietary differences.^18^ These inconsistencies may be due to differences in activity behaviour measurement (Fitbit vs. ActiGraph accelerometry), different participant pools or different measurement periods (i.e., 9‐day vs. continuous assessment).

While those studies^17^, ^18^ provide some insight into children's obesogenic behaviours in summer holidays versus the school year, several limitations should be noted. The findings from studies of low‐income, minority children may not be generalizable to the population and the small sample sizes for children (*n* = 30) and schools (*n* = 1–3) limit the ability to draw confident conclusions. Further, studies have predominantly been conducted in the United States (75% or 3/4). Data from other regions are needed to understand the impact of summer holidays on children's obesogenic behaviours around the globe. The purpose of this study was to examine differences in obesogenic behaviours during the summer holidays versus the school year in a sample of primary‐school‐aged Australian children using a within‐subject study design.

## 2 METHODS

### 2.1 Study sample and design

This study used data from the *Life on Holidays* project, for which the full methods have been previously published.^19^ Life on Holidays is a longitudinal cohort study that examines rates of change in fitness and fatness during the summer holidays and school year, and how rates of change in these outcomes relate to changes in activity and diet.^19^ This study presents activity and diet behaviour data from summer holidays and school years. Twenty‐four primary schools (21% uptake) located in different socioeconomic areas across metropolitan Adelaide, Australia, were recruited. From these schools, 381 participants (43% uptake) were recruited, though 23 did not commence baseline assessments (*n* = 1 moved school; *n* = 1 ineligible; *n* = 21 unknown reason), resulting in a final baseline sample size of 358 grade 4 children. Recruitment occurred in two waves (Wave 1: 2019 and Wave 2: 2020). Ethical approval was obtained from University of South Australia Human Research Ethics Committee (200980), the South Australian Department of Education and Child Development (2008–0055) and the Adelaide Catholic Education Centre (201820). Written informed parent consent and child assent were obtained.

### 2.2 Measurements

Measurements commenced in Term 1 of 2019 (wave 1) and Term 1 of 2020 (wave 2). Activity and diet were measured at five timepoints over 2 years to capture school and summer holiday values: Term 1 (February–March) of Grade 4, Term 4 (October–November) of Grade 4, summer holidays, Term 1 of Grade 5 and Term 4 of Grade 5. COVID‐19 impacted in‐school measurements during 2020 due to some schools not allowing external visitors on site. This impacted data collection for wave 1 participants (Term 12020: 1 school [10 children]; Term 42020: 3 schools [48 children]) and for wave 2 participants (Term 12020: 1 school [22 children]; Term 42020: 1 school [4 children]). This resulted in missing weight, fitness and accelerometry data for these children at these timepoints.

### 2.3 Activity behaviours

Children's time spent sleeping, sitting and in LPA and MVPA were captured using wrist‐worn accelerometers (GENEActiv; Activinsights, Cambridgeshire, UK). Accelerometers were worn for 24 h/day for 7 consecutive days at each timepoint. For assessments during the school year, a trained research assistant distributed and collected the accelerometers from the children's schools at the beginning and end of each 7‐day wear period. For assessments during the summer holidays, accelerometers were distributed to the children's schools in the last week of the school year, with reply‐paid envelopes, for children to start wearing on the first day of the summer holidays. Participants also recorded their sleep and wake times and periods of device removal along with reasons on a paper‐based form. Data were collected at 50 Hz and the epoch was set at 60‐s intervals. Validated cut‐points^20^ were used to identify durations of sedentary time and light‐ and moderate‐vigorous physical activity. Sleep duration was determined using the algorithm proposed by van Hees et al.^21^ Participants' accelerometry data were analysed if they wore the accelerometer for at least 10 waking hours, on at least 4 days, including at least three weekdays and one weekend day. The GENEActiv has strong intra‐instrument and inter‐instrument reliabilities (CV_intra_ = 1.4% and CV_inter_ = 2.1%), very good test–retest reliability (ICC = 0.67–0.87)^22^ and excellent convergent validity (*r* = 0.98).^23^

Specific types of activity were self‐reported by children using the Multimedia Activity Recall for Children and Adolescents (MARCA). The MARCA is a computerized 24‐h recall in which participating children recalled everything they did over the previous 2 days, using a segmented day format with a resolution of 5 min or more. Recalls occurred during four separate 30‐min face‐to‐face interviews (school time), and once during a computer‐assisted telephone interview (summer holidays). The 259 individual activities in the MARCA are hierarchically aggregated into eight ‘Superdomains’, Domestic/Social, Passive Transport, Physical Activity, Quiet Time, School‐Related, Screen Time, Self‐Care and Sleep. The MARCA has established validity (*r* = 0.4–0.7) when compared against accelerometry,^24^ pedometry^25^ and doubly labelled water,^26^ as well as excellent test–retest reliability (ICC = 0.88–1.00).^24^

### 2.4 Diet

Child diet was reported by parents using the Automated Self‐Administered 24‐h Dietary Assessment Tool (ASA24), Australian version (2016), developed by the National Cancer Institute, Bethesda, MD, USA.^27^ The ASA24 is an online tool for collecting 24‐h dietary recall data using the seven‐pass method (i.e., meal‐based quick list, meal gap review, detail pass, forgotten foods, final review, last chance, usual intake) with digital photographic measures to aid portion size estimation.^28^, ^29^ Recalls were administered via phone by a trained research assistant. Parent/carers completed the recalls as proxy, with the child present where possible, recalling food and drinks consumed over the previous 24 h. Food group intake, energy and nutrient intake was estimated using the Australian Food Supplement and Nutrient Database (AUSNUT) 2011–2013.^30^ Diet quality was assessed using the Dietary Guidelines Index for Children and Adolescents (DGI‐CA), which provides a measure of adherence to the Australian Dietary Guidelines by children and adolescents.^31^ Eleven indicators, which reflect diet variety, adequacy, quality and moderation, are combined to provide a score from 0 to 100, with lower scores indicating poorer dietary guideline compliance.

### 2.5 Anthropometric assessment

Height and weight were obtained using a Seca 213 stadiometer (Seca, Hamburg, Germany) and InBody BIA scales (InBody USA, Cerritos, CFA). Body mass index (BMI) was calculated as weight in kilograms divided by the square of height in metres (kg/m^2^) and converted to BMI *z*‐scores using the World Health Organization child growth standards.^32^ The International Obesity Taskforce Criteria were used to categorize children as thin, normal weight, overweight or obese.^33^, ^34^

### 2.6 Socioeconomic position

Socioeconomic position (SEP) was reported by parents in a demographic questionnaire at baseline. Both parents reported their occupation, household income and highest education level. From these, a composite SEP *z*‐score was derived and categorized as low, middle and high SEP, based on the procedure outlined in Gibbings et al.^35^

### 2.7 Analyses

Analyses were performed using Stata (v. 17.0, College Station, TX). Only children with valid data from at least two in‐school timepoints and the summer holiday timepoint, as well as complete SEP, sex, BMI category and pubertal status data were included for analyses. Mixed‐effects models were used to assess differences in activity and diet between the summer holidays and school years. Analyses were adjusted for participants nested in schools and in waves. Interactions between time (school vs. summer holidays) and subgroups (i.e., SEP, sex, pubertal status and BMI) were also explored. For significant interactions, modelled differences between school and summer holidays for each subgroup were calculated. Results from Stata's equality of standard deviation (variance) test using the *sdtest* command indicated that the assumption of homogeneity of variance was violated—variance was greater during holidays than during the in‐school periods. Stata's *residuals* (*independent, by*(*timepoint*)) command was used to accommodate the heteroscedasticity induced by the timepoint variable. The alpha level (0.05) was adjusted for multiple comparisons using Holm–Bonferroni adjustment, with adjustments made separately for MARCA, GENEActiv and diet assessments. No adjustments for multiple comparisons were carried out for the subgroup analyses (i.e., SEP, sex, pubertal status and BMI). Statistical significance was set at *p* < 0.05.

## 3 RESULTS

There were different subsets of children included in each analyses. One hundred and sixty nine children had complete demographic data AND at least one of the following: complete MARCA (*n* = 133); complete GENE (*n* = 133) and/or complete diet data (*n* = 128). Table 1 provides the demographic characteristics of participants. Additionally, characteristics of participants excluded from analyses (due to missing data for accelerometry, MARCA, diet or demographic data) are presented. Compared with included children, excluded children were similar in age and BMI category, but a greater proportion were male, and from lower SEP households, and slightly more were classified as early pubertal and slightly fewer as mid + pubertal.

**TABLE 1:** Demographic characteristics of participants.

	Included in analyses	Excluded from analyses	*p* (*t*‐test/*χ* ^2^)
*N*	169	212	
Age at baseline; mean (SD)	9.4 (0.3)	9.4 (0.4)	0.30
Sex; *n* (%) male	62 (37)	99 (47)	**0.045**
BMI category; *n* (%)		*n* = 131	0.468
Normal weight	130 (77)	96 (73)	
Overweight/obese	39 (23)	35 (27)	
SEP tertile		*n* = 127	**0.024**
Low	38 (22)	47 (37)	
Middle	82 (49)	51 (40)	
High	49 (29)	29 (23)	
Pubertal status		*n* = 113	**0.031**
Pre‐pubertal	103 (61)	63 (56)	
Early pubertal	34 (20)	37 (33)	
Mid + pubertal	32 (19)	13 (12)	

Abbreviations: BMI, body mass index; SEP, socioeconomic position.

*Note*: Bold values indicates *p* < 0.05.

Table 2 shows the mean school and summer holiday values for activity and diet behaviours, along with the results of the mixed‐effects model. Children spent less time in school‐related activities during the summer holidays, compensated for by spending more time on screens, and in domestic/social, self‐care, passive transport and quiet time activities. Children spent more time in sedentary behaviours, slept less and had lower MVPA and total counts during the summer holidays compared with the school year. Diet quality was lower, and children consumed fewer serves of fruit during the summer holidays, compared with the school year.

**TABLE 2:** Activity and diet behaviours in summer holidays relative to school time.^a^

	School				Summer holidays				Mixed‐effects model *β* (95% CI)	*p*‐value
	Mean	SD	Min	Max	Mean	SD	Min	Max		
MARCA^b^ (*n* = 133)										
Domestic/social	**61**	**42**	**1**	**228**	**105**	**95**	**0**	**480**	**45 (30, 59)**	0.000*
Screen	**186**	**96**	**20**	**517**	**259**	**141**	**0**	**713**	**73 (51, 94)**	0.000*
School‐related	**218**	**94**	**3**	**423**	**53**	**67**	**0**	**393**	**−165 (−181, −149)**	0.000*
Self‐care	**107**	**20**	**65**	**171**	**130**	**38**	**35**	**230**	**23 (16, 30)**	0.000*
Passive transport	**37**	**23**	**0**	**143**	**59**	**74**	**0**	**725**	**22 (9, 35)**	0.001*
Quiet time	**60**	**29**	**16**	**184**	**76**	**74**	**0**	**325**	**16 (3, 29)**	0.016*
Physical activity	148	60	36	370	131	100	0	480	−17 (−35, 0.9)	0.063
Sleep	627	87	388	857	623	52	430	1140	4 (−12, 20)	0.648
GENEActiv^c^ (*n* = 133)										
Sleep	**563**	**28**	**503**	**632**	**550**	**42**	**442**	**644**	**−13 (−19, −7)**	0.000*
Sedentary	**513**	**69**	**354**	**741**	**540**	**96**	**352**	**823**	**27 (15, 39)**	0.000*
LPA	288	50	152	418	284	75	118	484	−3 (−12, 6)	0.481
MVPA	**79**	**28**	**29**	**154**	**68**	**44**	**3**	**306**	**−12 (−19, −5)**	0.001*
Total counts	**272 219**	**45 306**	**162 125**	**38 495**	**235 729**	**50 244**	**117 370**	**378 212**	**−36 491 (−43 834, −29 148)**	0.000*
Diet^d^ (*n* = 128)										
Energy (kJ)	8142	1725	4576	16 741	8205	2628	3821	17 705	64 (−416 544)	0.794
Protein (g)	77	24	38	231	76	27	19	156	−0.8 (−6, 5)	0.771
Total fat (g)	74	20	19	147	79	33	23	205	5 (−1, 12)	0.098
Carbohydrate (g)	234	53	127	480	228	77	100	512	−6 (−19, 8)	0.399
Fruit (# of serves)	**2**	**1**	**0**	**5**	**1**	**1**	**0**	**8**	**−0.4 (−0.7, −0.2)**	0.000*
Vegetables and legumes (# of serves)	3	2	0	11	2	2	0	11	−0.5 (−0.9, −0.1)	**0.018**
Grains (# of serves)	4	2	1	17	4	2	0	11	−0.4 (−0.9, 0.1)	0.122
Meats and alternatives (# of serves)	1	1	0	6	1	1	0	5	−0.07 (−0.3, 0.2)	0.554
Dairy (# of serves)	1	1	0.1	4	1	1	0	6	−0.05 (−0.3, 0.2)	0.657
Discretionary choices (# of serves)	5	2	0.5	14	6	4	0	22	0.6 (−0.1, 1.4)	0.097
Diet quality (/score 100)	**39**	**9**	14	59	**35**	**13**	0	70	**−4 (−6, −2)**	0.000*

*Note*: Bold text denotes statistical significance *p* < 0.05, with * denoting statistical significance after Holm–Bonferroni adjustment was applied. Activity values are in minutes per day, except for counts. Analyses adjusted for the nested sample design (observations nested within participants, within schools and within waves).

Abbreviations: BMI, body mass index; LPA, light‐intensity physical activity; MARCA, Multimedia Activity Recall for Children and Adolescents; MVPA, moderate‐to‐vigorous physical activity; SEP, socioeconomic position.

^a^ The comparator is the in school value.

^b^ Participants with complete MARCA data for holiday and in‐school measurements, sex, BMI, SEP (composite measure) and pubertal status.

^c^ Participants with complete GENE data for holiday and in‐school measurements, sex, BMI, SEP and pubertal status.

^d^ Participants with complete diet data for holiday and in‐school measurements, sex, BMI, SEP and pubertal status.

Supplementary Tables 1–4 show the observed differences between activity and diet behaviours in the summer holidays compared with the school year for each of the different subgroups (i.e., sex, SEP, BMI and pubertal status). Where interactions between time (school vs. summer holidays) and subgroups were significant, modelled differences between school and summer holidays were calculated separately for each subgroup and are described below.

The interaction between time and weight status was significant for LPA (27; 95% CI: 6, 48), screen time (−65; 95% CI: −119, −11) sedentary time (−35; 95% CI: −62, −7) and number of meat serves (−0.6; 95% CI: −1, −0.2). Children classified as overweight/obese engaged in more LPA (modelled difference = +6 min/day), more screen time (modelled difference = +46 min/day), less sedentary time (modelled difference = −2 min/day) and consumed fewer serves of meat (modelled difference = −0.3 serves/day) in the summer holidays compared with the school year. In contrast, children classified as normal weight engaged in less LPA (modelled difference = −21 min/day), more screen time (modelled difference = +111 min/day), more sedentary time (modelled difference = +37 min/day) and consumed more serves of meat (modelled difference = +0.3 serves/day) in the summer holidays compared with the school year.

The interaction between time and SEP was significant for quiet time (44; 95% CI: 7, 80), school‐related time (−46; 95% CI: −91, −1) and MVPA (−21; 95% CI: −39, −4). Children from high‐SEP backgrounds engaged in more quiet time (modelled difference = +41 min/day) and less school‐related time (modelled difference = −180 min/day) in the summer holidays compared with the school year. In contrast, children from low‐SEP backgrounds engaged in less quiet time (modelled difference = −3 min/day) and less school‐related time (modelled difference = −134 min/day) in the summer holidays compared with the school year. Children from middle‐SEP (modelled difference = −22 min/day) and high‐SEP backgrounds (modelled difference = −1.0 min/day) engaged in less MVPA in the summer holidays compared with the school year.

The interaction between time and sex was significant for self‐reported sleep (40; 95% CI: 6, 74), GENEActiv counts (−25 047; 95% CI: 6526, 40 567) and serves of fruit (−0.7; 95% CI: −1, −0.2). Females reported more sleep (modelled difference = +6 min/day), had lower GENEActiv counts (modelled difference = −29 405 counts/day) and consumed less fruit (modelled difference = −0.9 serves/day) in the summer holidays compared with the school year. In contrast, males reported less sleep (modelled difference = −34 min/day), had lower GENEActiv counts (modelled difference = −54 452 counts/day) and consumed less fruit (modelled difference = −0.2 serves/day) in the summer holidays compared with the school year.

The interaction between time and pubertal status was significant for carbohydrate intake (40; 95% CI: 6, 73). Early pubertal children consumed more carbohydrates (modelled difference = +21 g/day), whereas pre‐pubertal children consumed less carbohydrates (modelled difference = −19 g/day) in the summer holidays compared with the school year.

## 4 DISCUSSION

### 4.1 Main findings

This study investigated within‐child differences in activity and diet between the school year and summer holidays and explored whether differences were moderated by sex, SEP, pubertal status or BMI status. Our study found that in the summer holidays, children spent less time in MVPA, more time sedentary, engaged in higher amounts of screentime, slept less, consumed fewer serves of fruit and their diet quality was poorer. Children spent less time in school‐related activities in the summer holidays and compensated for this by spending more time in domestic/social activities, self‐care, passive transport and quiet time. There were few differences in activity and diet behaviours in the summer holidays across sociodemographic characteristics.

### 4.2 Implications

The findings of the current study align with previous studies that have found children spent more time on screens during the summer holidays compared with the school year.^14^, ^16^, ^17^, ^18^, ^36^ This finding is expected given the presence of the 6‐h school day, which limits recreational screen time opportunities to mainly before and after school and on weekends. With the exception of school‐related activities, the largest difference in activity behaviours between the summer holidays and school year was for screen time—children spent 39% more time using screens during the summer holidays than during the school year. Modelled differences by weight category indicate this difference was even larger among children classified as normal weight (+111 min/day [+49%]) compared with children classified as overweight/obese (+46 min/day [+8%]). However, it should be noted that the sample in the overweight/obese group was small (*n* = 26), resulting in reduced statistical power. Nonetheless, increased screen time during the summer holidays carries several important implications. For example, meta‐analytic results indicate a 1‐h/day increment in TV viewing corresponded to a 13% increased risk of obesity.^37^ This, combined with the health and obesity risks associated with recent findings indicating that increases in fatness in children are occurring during the summer holidays and stagnating when children are in school,^1^, ^2^, ^3^, ^4^ provides evidence for the need for intervention targeting screen time in the summer holidays.

Children spent less time per day in MVPA (−12 min/day) and more time per day sedentary (+27 min/day) during the summer holidays compared with the school year. This finding is expected given the optimal temperature for high levels of MVPA (and low levels of sedentary time) was reported to be between 20 and 25°C.^38^ The average daily maximum summer temperature in summer in Adelaide is 28–29°C, perhaps limiting opportunities for outdoor physical activity (particularly given that maximum daily summer temperatures are highly variable, and temperatures exceeding 35°C and even 40°C are not uncommon). These results align with results from previous studies, which report reductions in MVPA of between 3 and 12 min/day and increases in sedentary time of between 24 and 28 min/day during the summer holidays compared with the school year.^15^, ^16^, ^18^

MVPA differed between SEP groups in interaction analyses with middle‐SEP children engaging in less MVPA (modelled difference: −22 min/day [22%]) and low‐SEP children engaging in more MVPA (modelled difference: −1.0 min/day [7%]) in the summer holidays compared with the school year. Contrary to our findings, studies have reported larger reductions in MVPA during the summer holidays among children eligible for free/reduced price lunch (i.e., low SEP), compared with those not eligible^16^ or no differences in MVPA during the summer holidays among children predominantly from low‐income, minority backgrounds.^17^ However, our low‐SEP group was comprised of only 26 children (20%), limiting statistical power. In contrast, the low‐SEP group in the study by Hunt et al.^16^ was comprised of 120 children (44% of the sample) and the sample in the study by Brazendale et al.^17^ was comprised of 30 children from low‐income, minority backgrounds, providing a possible explanation for the divergent results seen here.

There was no variation in the change in MVPA by weight status, but change in sedentary time in the summer holidays and school year was significantly different in time × weight category analyses. Modelled differences were relatively minimal (normal weight: +37 min/day [+7%]; overweight/obese: −2 min/day [−0.4%]). Thus, declines in MVPA and increases in sedentary time in the summer holidays appear to occur across all weight categories. Similarly, Volmut et al.^15^ reported that that changes in MVPA and sedentary time over the summer holidays were not associated with anthropometric measures.

In contrast to previous studies reporting children sleep longer in the summer holidays,^16^, ^17^ our findings indicate that device‐measured sleep was lower in the summer holidays. However, this difference was relatively small (−13 min/day) and on average children met the sleep guidelines of 9–11 h/night both during the summer holidays and school year, so the summer holiday versus school time difference is unlikely to be clinically significant. Change in reported sleep in the summer holidays and school year was significantly different in time × sex analyses; females reported more sleep (modelled difference = +6 min/day), whereas males reported less sleep (modelled difference = −34 min/day) in the summer holidays compared with the school year. However, there were no sex differences in device‐measured sleep duration between summer holidays and school year, and modelled differences in self‐reported sleep duration were minimal. To our knowledge, no other study has compared sex differences in sleep patterns in the summer holiday versus school year. Thus, due to limited literature combined with divergent results for device‐measured versus self‐reported sleep duration, it is difficult to draw confident conclusions here.

Children had poorer diet quality in the summer holidays; however, there was no notable or significant change in energy intake. These findings are consistent with studies reporting children consumed less fruit and vegetables and more added sugars during the summer holidays versus school time,^14^ but are in contrast with studies concluding no dietary differences^18^, ^36^ or that children consumed more fruit during the summer holidays.^17^ The higher diet quality during school time is consistent with common school nutrition policy promoting low energy density foods such as fruit and vegetables. The lack of a difference in energy intake, given the lower MVPA and higher sedentary time, signals a potential risk of positive energy balance in the summer holidays. Overall, the literature on children's dietary intake during the summer holidays is inconsistent, perhaps partly due to considerable heterogeneity in the structure of summer holidays across different countries, participant pools, dietary assessment tools and dietary components assessed. For example, studies assessed vegetable and added sugar consumption,^14^ fruits, vegetables, dairy, convenience foods, sweets and desserts, and sugar‐sweetened beverages consumption,^17^ or dichotomized healthy (e.g., fruits, vegetables, unsweetened dairy, water) and unhealthy foods and drinks (e.g., convenience foods, sweets/desserts, sugar‐sweetened beverages).^18^ For these reasons, it is difficult confidently compare conclusions regarding children's dietary intake during the summer holidays versus school year.

Findings from this study suggest that the school environment shapes healthier diet and activity behaviours, particularly screen time. The Structured Days Hypothesis (SDH) posits that more unhealthy obesogenic behaviours may occur over the summer holidays due to the absence of the structure of the school day (e.g., adult supervision, routine opportunities for physical activity, set lunch and snack times).^39^ Thus, structured summer holiday programming may provide an efficacious strategy for mitigating accelerated BMI gain in the summer holidays, through more favourable activity behaviours. Previous studies have reported success, finding that on summer days children engage in more MVPA, have higher energy expenditure, less sedentary time and have more consistent sleep schedules when they attend a structured programme (e.g., summer camp or summer day program) compared with days they do not attend.^40^, ^41^, ^42^

The SDH also provides explanation for the observed changes in children's movement behaviours during home quarantine/lockdown imposed by the COVID‐19 pandemic, with school closures inadvertently demonstrating the importance of structure in shaping children's movement behaviours and weight‐related outcomes.^43^, ^44^ For example, Okely et al.^44^ showed that children spent 55 min/day more time sedentary, with later bed and wake times compared with before the COVID‐19 pandemic.

### 4.3 Strengths and limitations

This is the first Australian study to compare children's activity and diet patterns in the summer holidays and school year. We recruited a representative sample of children across socioeconomic groups, and we collected data on all activity and diet behaviours of the same children in both the school year and summer holidays, allowing within‐person comparisons. We also utilized valid and reliable measurements of activity and diet.

This study has limitations that should be considered when interpreting the findings. Data were missing because the COVID‐19 pandemic commenced approximately halfway through data collection, with some schools not allowing further data collection. The COVID‐19 pandemic also likely contributed to participant dropout, which was considerable. The included and excluded sample were broadly similar in age and BMI status, but differed in terms of SEP, pubertal status and sex. Another limitation was the lack of information on the context of children's days during summer vacation, such as whether they attended structured programming. Based on the SDH, children who attended structured programming such as vacation care may not experience unhealthy changes in obesogenic behaviours on those days. Future studies should track children's attendance at structured summer programming. The narrow age range of participants (age 9–11 years) and narrow geographical location (all participants were recruited from one Australian city) were further limitations.

## 5 CONCLUSION

In summary, this study provides some evidence that children engage in less healthful activity and diet behaviours in the summer holidays, compared with the school year. These findings are consistent with the SDH, suggesting that the school day may regulate children's obesogenic behaviours and the absence of school may cause increased unhealthy behaviours. We recommend that screen time be a target for intervention in the summer holidays.

## CONFLICT OF INTEREST STATEMENT

No conflict of interest was declared.

## Supporting information


**Supplementary Table 1:** Activity and diet behaviours in summer holidays relative to school time reported separately for boys and girls.
**Supplementary Table 2:** Activity and diet behaviours in summer holidays relative to school time reported separately for low‐, middle‐ and high‐socioeconomic position bands.
**Supplementary Table 3:** Activity and diet behaviours in summer holidays relative to school time reported separately for normal weight and overweight/obese categories.
**Supplementary Table 4:** Activity and diet behaviours in summer holidays relative to school time reported separately for pubertal categories.
