# Supplementary material for: Children's activity and diet behaviours in the summer holidays versus school year
Source: Pediatr Obes. 2023 Mar 21;18(7):e13029. doi: 10.1111/ijpo.13029 (PMC10909460; doi:10.1111/ijpo.13029)
Supplement: Supplementary file 1 — Supplementary Table 1: Activity and diet behaviours in summer holidays relative to school time reported separately for boys and girls. Supplementary Table 2: Activity and diet behaviours in summer holidays relative to school time reported separately for low‐, middle‐ and high‐socioeconomic position bands. Supplementary Table 3: Activity and diet behaviours in summer holidays relative to school time reported separately for normal weight and overweight/obese categories. Supplementary Table 4: Activity and diet behaviours in summer holidays relative to school time reported separately for pubertal categories. [file IJPO-18-e13029-s001.pdf]

Title:

Children’s activity and diet behaviours in the summer holidays versus school year

Authors:

Amanda WATSON, University of South Australia, School of Health Sciences, GPO Box 2471, Adelaide SA 5001, [amanda.watson@unisa.edu.au]

Carol MAHER, University of South Australia, School of Health Sciences, GPO Box 2471, Adelaide SA 5001, [Carol.Maher@unisa.edu.au]

Rebecca Golley, Flinders University, College of Nursing and Health Sciences, Adelaide SA 5001, [rebecca.golley@flinders.edu.au]

Dot Dumuid, University of South Australia, School of Health Sciences, GPO Box 2471, Adelaide SA 5001, [Dot.Dumuid@unisa.edu.au]

Alexandra MANSON, Flinders University, College of Nursing and Health Sciences, Adelaide SA 5001, [alexandra.manson@flinders.edu.au]

Grant Tomkinson, University of South Australia, School of Health Sciences, GPO Box 2471, Adelaide SA 5001, [grant.tomkinson@unisa.edu.au]

Francois Fraysse, University of South Australia, School of Health Sciences, GPO Box 2471, Adelaide SA 5001, [Francois.Fraysse@unisa.edu.au]

Tim OLDS, University of South Australia, School of Health Sciences, GPO Box 2471, Adelaide SA 5001, [Timothy.Olds@unisa.edu.au]

Supplementary Table 1: Activity and diet behaviours in summer holidays relative to school time reported separately for boys and girls

	School				Summer holidays				p-value
	Boys (n=47)		Girls (n=86)		Boys (n=47)		Girls (n=86)		
**MARCA**	**Mean**	**SD**	**Mean**	**SD**	**Mean**	**SD**	**Mean**	**SD**	
Domestic/social	54	44	64	40	100	99	108	94	0.712
Screen	214	107	171	87	308	153	232	127	0.370
Physical activity	171	80	136	42	145	105	124	97	0.370
School-related	185	91	235	91	41	65	59	67	0.111
Self-care	101	21	111	19	128	40	131	37	0.389
Passive transport	40	22	36	23	55	44	61	86	0.586
Quiet time	54	28	63	29	70	62	79	79	0.829
Sleep	**621**	**64**	**624**	**44**	**593**	**67**	**645**	**91**	**0.021**
**GENEActiv**	**Boys (n=46)**		**Girls (n=87)**		**Boys (n=46)**		**Girls (n=87)**		
Sleep	561	30	564	28	548	41	552	43	0.753
Sedentary	513	66	513	71	552	98	533	95	0.142
LPA	276	44	294	53	265	74	295	74	0.361
MVPA	92	29	73	25	79	41	62	45	0.729
Total counts	293147	43903	261154	42236	238603	45335	234208	54845	0.002
**Diet**	**Boys (n=45)**		**Girls (n=83)**		**Boys (n=45)**		**Girls (n=83)**		
Energy (kJ)	8634	2046	7875	1469	8789	2424	7889	2693	0.957
Protein (g)	86	31	72	18	84	32	72	23	0.556
Total fat (g)	73	22	74	19	84	32	77	33	0.321
Carbohydrate (g)	254	61	223	44	242	71	220	80	0.420
Fruit (# of serves)	**2**	**1**	**2**	**1**	**2**	**2**	**1**	**1**	**0.008**
Vegetables & legumes (# of serves)	3	2	2	2	2	2	2	2	0.556

Grains (# of serves)	5	2	4	1	4	2	4	2	0.104
Meats & alternatives (# of serves)	2	1	1	1	2	1	1	1	0.885
Dairy (# of serves)	2	1	1	1	2	1	1	1	0.941
Discretionary choices (# of serves)	5	3	5	2	6	3	6	4	0.505
Diet quality (/score 100)	41	8	37	9	37	14	34	12	1.000

Bold text denotes statistical significance $p<0.05$
Activity values are in min/day, except for counts.

Supplementary Table 2: Activity and diet behaviours in summer holidays relative to school time reported separately for low-, middle- and high-SEP bands

	School						Summer holidays						p-value mid vs. low	p-value high vs. low
	Low (n=26)		Middle (n=65)		High (n=42)		Low (n=26)		Middle (n=65)		High (n=42)			
**MARCA**	**Mean**	**SD**	**Mean**	**SD**	**Mean**	**SD**	**Mean**	**SD**	**Mean**	**SD**	**Mean**	**SD**		
Domestic/social	61	42	63	38	57	47	93	84	116	104	97	86	0.303	0.621
Screen	223	76	192	102	154	91	296	122	274	154	213	120	0.934	0.532
Physical activity	136	50	157	62	142	62	128	110	124	96	145	100	0.340	0.742
School-related	**197**	**78**	196	96	**264**	**84**	**47**	**81**	57	58	**65**	**69**	0.877	**0.046**
Self-care	98	18	110	20	108	20	125	49	128	34	138	37	0.429	0.687
Passive transport	34	19	41	25	34	19	69	140	51	39	65	56	0.210	0.949
Quiet time	**59**	**25**	62	32	**56**	**27**	**58**	**70**	69	69	**97**	**80**	0.616	**0.019**
Sleep	632	76	618	43	625	47	624	128	632	81	621	63	0.387	0.982
**GENEActiv**	**Low (n=28)**		**Middle (n=67)**		**High (n=38)**		**Low (n=28)**		**Middle (n=67)**		**High (n=38)**			
Sleep	550	29	568	27	564	28	539	551	557	39	547	39	0.960	0.573
Sedentary	536	75	507	62	505	73	559	110	546	88	521	100	0.163	0.970
LPA	282	57	290	47	289	52	275	89	279	72	302	68	0.943	0.084
MVPA	**73**	**23**	**78**	**29**	85	29	**78**	**60**	**61**	**39**	71	38	**0.016**	0.068
Total counts	264665	42557	271159	43674	279665	49970	233408	56979	229413	44931	248573	52822	0.317	0.962
**Diet**	**Low (n=21)**		**Middle (n=67)**		**High (n=40)**		**Low (n=21)**		**Middle (n=67)**		**High (n=40)**			
Energy (kJ)	7989	1632	8425	8107	7746	1578	8312	3300	8025	2614	8453	2283	0.347	0.458
Protein (g)	72	25	80	26	75	20	79	35	75	25	76	25	0.138	0.501
Total fat (g)	74	24	77	19	69	19	81	41	77	20	82	31	0.482	0.419
Carbohydrate (g)	231	38	241	56	223	54	230	89	224	80	235	65	0.527	0.339
Fruit (# of serves)	1	1	2	1	2	1	1	1	1	1	2	2	0.886	0.191

Vegetables & legumes (# of serves)	2	1	3	2	3	1	1	1	2	2	3	2	0.468	0.442
Grains (# of serves)	4	1	4	2	4	2	4	2	4	3	4	2	0.467	0.424
Meats & alternatives (# of serves)	1	1	1	1	1	1	1	1	1	1	1	1	0.308	0.324
Dairy (# of serves)	1	1	1	1	2	1	1	1	1	1	2	1	0.197	0.299
Discretionary choices (# of serves)	6	2	6	2	4	2	7	6	5	3	6	3	0.792	0.098
Diet quality (/score 100)	33	9	39	7	41	9	30	14	35	13	37	13	0.750	0.512

Bold text denotes statistical significance p<0.05
Activity values are in min/day, except for counts.

Supplementary Table 3: Activity and diet behaviours in summer holidays relative to school time reported separately for normal weight and overweight/obese categories

	School				Summer holidays				p-value
	Normal weight (n=107)		Overweight/obese (n=26)		Normal weight (n=107)		Overweight/obese (n=26)		
**MARCA**	**Mean**	**SD**	**Mean**	**SD**	**Mean**	**SD**	**Mean**	**SD**	
Domestic/social	60	42	63	40	110	98	88	83	0.168
Screen	**179**	**98**	**215**	**85**	**266**	**148**	**232**	**104**	**0.019**
Physical activity	151	62	138	49	126	92	153	127	0.074
School-related	220	96	207	84	56	71	39	41	0.989
Self-care	108	20	102	18	128	39	138	37	0.075
Passive transport	36	23	40	20	53	47	82	136	0.190
Quiet time	60	30	59	27	74	70	83	89	0.467
Sleep	625	49	615	64	627	86	625	95	0.974
**GENEActiv**	**Normal weight (n=103)**		**Overweight/obese (n=30)**		**Normal weight (n=103)**		**Overweight/obese (n=30)**		
Sleep	565	29	557	27	551	42	547	43	0.643
Sedentary	**514**	**72**	**509**	**57**	**549**	**101**	**507**	**71**	**0.014**
LPA	**283**	**51**	**304**	**45**	**274**	**74**	**321**	**67**	**0.013**
MVPA	81	28	73	26	68	41	67	54	0.689
Total counts	273883	47050	266506	38900	233595	50053	243053	51062	0.084
**Diet**	**Normal weight (n=102)**		**Overweight/obese (n=26)**		**Normal weight (n=102)**		**Overweight/obese (n=26)**		
Energy (kJ)	8147	1774	8121	1548	8070	2508	8736	3047	0.344
Protein (g)	77	25	79	21	76	26	79	29	0.970
Total fat (g)	74	20	75	21	77	31	86	39	0.353
Carbohydrate (g)	235	55	229	45	225	74	240	88	0.337
Fruit (# of serves)	2	1	2	1	1	1	1	1	0.694

Vegetables & legumes (# of serves)	3	2	3	1	2	2	2	2	0.602
Grains (# of serves)	4	2	4	2	4	2	4	3	0.720
Meats & alternatives (# of serves)	**1**	**1**	**2**	**1**	**1**	**1**	**1**	**1**	**0.042**
Dairy (# of serves)	2	1	1	1	2	1	1	1	0.542
Discretionary choices (# of serves)	**5**	**2**	**5**	**2**	**5**	**3**	**7**	**5**	**0.048**
Diet quality (/score 100)	39	9	37	9	36	13	32	13	0.507

Bold text denotes statistical significance $p<0.05$
Activity values are in min/day, except for counts.

Supplementary Table 4: Activity and diet behaviours in summer holidays relative to school time reported separately for pubertal categories

	School						Summer holidays						p-value pre- vs. early pubertal	p-value pre- vs. mid pubertal+
	Pre-pubertal (n=80)		Early pubertal (n=29)		Mid pubertal+ (n=24)		Pre-pubertal (n=80)		Early pubertal (n=29)		Mid pubertal+ (n=24)			
**MARCA**	**Mean**	**SD**	**Mean**	**SD**	**Mean**	**SD**	**Mean**	**SD**	**Mean**	**SD**	**Mean**	**SD**		
Domestic/social	53	38	78	47	65	40	91	84	147	102	105	109	0.057	0.661
Screen	189	95	191	94	173	108	274	151	244	129	228	116	0.401	0.754
Physical activity	149	64	145	64	148	39	137	98	123	90	124	119	0.500	0.333
School-related	226	90	185	92	229	103	62	77	32	39	47	47	0.562	0.608
Self-care	108	22	105	15	105	20	134	39	124	39	127	37	0.532	0.799
Passive transport	36	20	38	20	40	33	54	45	71	50	62	145	0.436	0.919
Quiet time	57	32	65	24	61	26	75	65	79	92	74	80	0.928	0.934
Sleep	621	49	634	45	618	67	615	67	619	84	674	128	0.361	0.023
**GENEActiv**	**Pre-pubertal (n=80)**		**Early pubertal (n=25)**		**Mid pubertal+ (n=28)**		**Pre-pubertal (n=80)**		**Early pubertal (n=25)**		**Mid pubertal+ (n=28)**			
Sleep	566	28	556	27	562	32	554	42	541	46	548	40	0.711	0.736
Sedentary	508	67	510	64	529	78	539	100	526	79	554	101	0.652	0.697
LPA	286	50	304	52	280	50	280	74	301	74	281	80	0.913	0.983
MVPA	84	29	73	23	72	26	69	40	72	56	59	44	0.162	0.922
Total counts	279365	44725	270801	11474	253069	43754	235990	483956	437710	58595	227854	48099	0.298	0.556
**Diet**	**Pre-pubertal (n=79)**		**Early pubertal (n=27)**		**Mid pubertal+ (n=22)**		**Pre-pubertal (n=79)**		**Early pubertal (n=27)**		**Mid pubertal+ (n=22)**			
Energy (kJ)	8113	1929	8056	1481	8348	1184	8187	2169	9063	3706	7218	2303	0.097	0.088
Protein (g)	77	28	73	19	81	15	77	27	77	26	71	28	0.683	0.130
Total fat (g)	73	21	74	19	78	16	79	28	86	45	70	30	0.339	0.219

Carbohydrate (g)	**234**	**57**	**234**	**50**	232	40	**225**	**64**	**264**	**109**	195	57	**0.020**	0.085
Fruit (# of serves)	2	1	1	1	2	1	1	2	1	1	1	1	0.864	0.198
Vegetables & legumes (# of serves)	3	1	2	2	3	2	2	2	2	2	2	3	0.984	0.842
Grains (# of serves)	5	2	4	1	4	1	4	2	4	2	3	2	0.762	0.071
Meats & alternatives (# of serves)	1	1	1	1	2	1	1	1	1	1	1	1	0.440	0.964
Dairy (# of serves)	2	1	1	1	1	1	2	1	1	1	1	1	0.336	0.971
Discretionary choices (# of serves)	5	2	6	3	6	2	5	3	8	6	5	4	0.110	0.413
Diet quality (/score 100)	39	8	37	10	38	9	36	12	32	15	36	12	0.674	0.757

Bold text denotes statistical significance p<0.05

Activity values are in min/day, except for counts.
